# Advanced Proteomics as a Powerful Tool for Studying Toxins of Human Bacterial Pathogens

**DOI:** 10.3390/toxins11100576

**Published:** 2019-10-04

**Authors:** Catherine Duport, Béatrice Alpha-Bazin, Jean Armengaud

**Affiliations:** 1 SQPOV, UMR0408, Avignon Université, INRA, F-84914 Avignon, France; 2 Laboratoire Innovations technologiques pour la Détection et le Diagnostic (Li2D), Service de Pharmacologie et Immunoanalyse (SPI), CEA, INRA, F-30207 Bagnols sur Cèze, France; beatrice.alpha-bazin@cea.fr (B.A.-B.); jean.armengaud@cea.fr (J.A.)

**Keywords:** bacterial toxins, proteomics, human pathogens, *B. cereus*

## Abstract

Exotoxins contribute to the infectious processes of many bacterial pathogens, mainly by causing host tissue damages. The production of exotoxins varies according to the bacterial species. Recent advances in proteomics revealed that pathogenic bacteria are capable of simultaneously producing more than a dozen exotoxins. Interestingly, these toxins may be subject to post-transcriptional modifications in response to environmental conditions. In this review, we give an outline of different bacterial exotoxins and their mechanism of action. We also report how proteomics contributed to immense progress in the study of toxinogenic potential of pathogenic bacteria over the last two decades.

## 1. Introduction

The first bacterial toxin, i.e., diphtheria toxin, was discovered in the culture filtrates of *Corynebacterium diphtheriae* in 1888. Within the next eight years, tetanus toxin and botulinum neurotoxin were detected in *Clostridium tetani* and *Clostridium botulinum* culture filtrates, respectively [1]. Since these pioneering works carried out at the beginning of the Pasteurian era, many gram-positive and gram-negative bacteria have been shown to secrete toxins. Importantly, many toxins are produced by human pathogenic bacteria.

Pathogenic bacteria display various levels of host specificity. Some bacteria require the human host as part of their life cycle, while many others have primary reservoirs in other animals. Some human pathogens are transmitted through food, while others are capable of being transmitted via several different routes [2,3]. Amongst the various virulence factors produced by pathogenic bacteria, toxins play an important role because they have an offensive role in causing tissue damage associated with many infectious diseases [4].

Toxins produced by pathogens can be divided into endotoxins and exotoxins. On the one hand, endotoxins are complex components of the outer membrane of Gram-negative bacteria. Structurally, they contain O-antigen, core polysaccharide, and toxic lipid A components [5]. Endotoxins are generally released during bacterial growth (due to rupturing of cell membrane), but they can be released after lysis of bacteria resulting from either autolysis or external lysis. Endotoxins act generally close to the infectious site and exhibit multiple injurious biological activities. They are very stable molecules that are capable of resisting extreme temperatures and pH values [6]. On the other hand, exotoxins are proteins secreted by both Gram-positive and Gram-negative bacteria. Compared to endotoxins, they are more specific. Since they are mostly secreted, they act at a site that can be distant from the infectious site. Some exotoxins are released only upon bacterial lysis [7]. Interestingly, exotoxins are often associated with foodborne outbreaks [8].

Since 1987, exotoxins have been amenable to crystallization and several three-dimensional crystalline structures have been established by high-resolution X-ray diffraction. This has contributed to the in-depth knowledge of the mechanisms of action of toxins and their classification into various families [9].

With the progress of molecular biology and then genomics, the structural genes of a large number of bacterial toxins and regulatory genes associated with their production have been identified for numerous pathogens. For the majority of the bacterial toxins studied to date, the genes are located on the main bacterial chromosome, highlighting their importance for the microorganism perpetuation. However, some are carried by extrachromosomal genetic elements (plasmids) and thus are potentially transmissible [10,11].

Bacterial toxins can be detected using various conventional methods including molecular biology techniques, such as polymerase chain reaction (PCR), and/or immunological techniques, such as enzyme-linked immunosorbent assays (ELISA) or western blotting. These methods are valuable for rapid preliminary screening but are associated with analytical limitations. Unequivocal detection and quantitation of toxins can be achieved using proteomics, which have gained in effectiveness over the last decade thank to the continuous development of mass spectrometry (MS) technologies (high resolution, accurate mass HR/AM instruments, hybrid configurations). In addition, proteomics provides information on cellular pathways that govern the production of toxins [12].

In this review, we focus on the current knowledge about the human bacterial exotoxins with a particular spotlight on the crucial contribution of proteomics in this area. In the first part, we give an overview of the bacterial exotoxin functional groups. In the second part, we emphasize the significant contribution of proteomics to detect exotoxins and their post-translational modifications.

## 2. Bacterial Exotoxins, the Key Arsenal of Pathogens

Bacterial exotoxins can be divided into four groups based on their modes of action [13]. These four groups, include (i) toxins that bind to the surface of target cell cytoplasmic membrane receptors and modify cell physiology by triggering intracellular signaling; (ii) toxins that bind to cell cytoplasmic membranes and disrupt the membrane lipid bilayer through expression of phospholipase activity or pore formation; (iii) AB toxins that are composed of two distinct molecular components, A and B. The B component binds to a specific receptor of the target cell and allows the component A to translocate into the cytoplasm. The A component is an enzyme that acts on a specific cytosolic target; and (iv) toxins with an enzymatic activity that enter the cytosol of the target cell via an injection apparatus. Although exotoxins may target different cell types, some of them specifically target intestinal cells producing typical food poisoning symptoms and causing diarrhea. These exotoxins are named enterotoxins [14].

### 2.1. Enterotoxins Modulating Signal Transduction Pathways

Enterotoxigenic *Escherichia coli* (ETEC) strains [15] secrete heat-labile (LT) and heat-stable (ST) enterotoxins that activate intracellular signaling pathways in the small intestinal epithelial cells, leading to disruption of the electrolyte homeostasis and finally provoke acute diarrhea in humans and animals [16]. LT enterotoxins are endocytosed after binding to host cell GM1 gangliosides in the gut and then trigger constitutive cAMP production. There are two subtypes of ST enterotoxins (STa and STb) that can be distinguished from each other by their solubility in methanol and their protease sensitivity. Both ST enterotoxin subtypes are first translated from plasmid located genes into propeptides, and then, these molecules are processed into mature toxins that properly fold outside the bacteria [17]. STa toxin binds to the guanylate cyclase C receptor and activates its intracellular catalytic domain, causing the hydrolysis of GTP and accumulation of intracellular cyclic GMP. STb toxin interacts specifically with sulfatide present on the surface of intestinal epithelial cells. This interaction activates a pertussis toxin-sensitive GTP-binding regulatory protein and subsequently causes accumulation of intracellular Ca^2+^ [18].

### 2.2. Membrane Damaging Toxins

Some exotoxins alter a critical structure of the plasma membrane, leading to the physical rupture thereof (cytolysis). These toxins are often called hemolysins because of the high sensitivity of erythrocytes to the lytic action of almost all these exotoxins. These mechanisms fall under two major processes.

In the first process, an enzymatic activity targets plasma membrane phospholipids. The first toxin recognized as possessing such enzymatic activity was the *Clostridium perfringens* α-toxin. This α-toxin has both a phospholipase C (PLC) and a sphingomyelinase (SMase) activity [19]. Other toxins with phospholipase activity include multifunctional autoprocessing repeats-in-toxin (MARTX) toxins of *Vibrio cholerae* [20] and adenylate cyclase toxins of *Bordetella pertussis*, which has a phospholipase A activity [21].

The second process involves transmembrane pore formation by Pore-Forming Toxins (PFTs). PFTs oligomerize upon binding to the eukaryotic plasma membrane and assemble into transmembrane stable pores that permeabilize cells to ions, metabolites, and proteins, triggering homeostasis problems in the host cell and a variety of coordinated host-cell responses. PFTs can be both necessary and sufficient for the pathogenesis of several bacterial species [22]. PFTs are categorized into two groups, α-PFT and, β-PFTs, based on the secondary structures used to form transmembrane pore domains [23]: α-PFTs form α-helical pores, while β-PFTs produce β-barrel pores.

**Figure 1:**
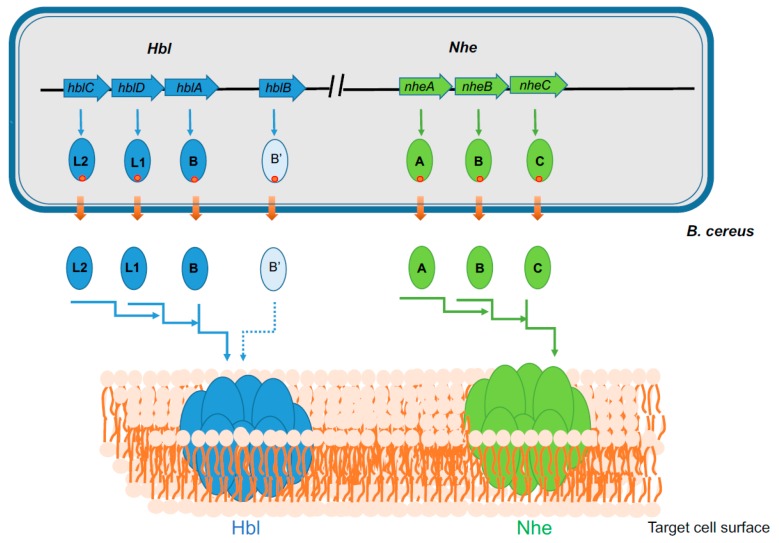
Genetic organization of *hbl* and *nhe* operons in *B. cereus* and schematic representation of the formation of Hbl and Nhe PFTs. Hbl operon comprises three ORFs, *hblC*DA that encode Hbl-L2, L1, and B proteins, respectively. Nhe operon comprises three ORFs, *nheABC*, that encode NheA, NheB, and NheC proteins, respectively. All the Hbl and Nhe components are synthesized as precursors with an N-terminal Sec pathway signal peptide (red circle). Transport through the bacterial membrane is accompanied by the release of both peptide signal and mature polypeptide in the extracellular media. The three mature components of both Hbl and Nhe bind sequentially to form PFTs in the plasma membrane of target eukaryotic cells. The *HblB* locus that encodes Hbl B’ is also shown. Its role in Hbl-PFT formation is currently unknown.

α-PFTs class includes the colicin and cytolysin A (ClyA) families. α-PFTs of the colicin-family are produced by *E. coli* strains [24,25]. α-PFTs of the ClyA family includes one-component toxins such as ClyA (also known as HlyE) of *E. coli* [26,27,28], two-component toxins [26,29], and three-component toxins such as the *Bacillus cereus* Non hemolytic enterotoxin (Nhe) and Hemolysin BL (Hbl) [30,31,32]. *B. cereus* Nhe consists of three proteins, NheA (41-kDa), NheB (39-kDa), NheC (40-kDa), encoded by one operon containing three ORFs, *nheA*, *nheB*, and *nheC*, respectively (Figure 1) [33]. Hbl is composed of the three components Hbl-L2 (46-kDa), Hbl-L1 (38-kDa), and Hbl-B (37-kDa), encoded by the *hbl*CDA operon [34] (Figure 1). The three Nhe components share sequence homology with one another and also with the three Hbl components. Studies have suggested that pore formation is due to sequential binding of Hbl-B, Hbl-L1, and Hbl-L2 and, NheC, NheB, and NheA for Hbl and Nhe, respectively [32,35,36]. Surprisingly, the crystal structure of NheA revealed it does not possess the hydrophobic β-tongue structure that is characteristic for Hbl-B and ClyA, suggesting a different mechanism of pore formation for Nhe [37,38].

β-PFT class comprises the hemolysin, aerolysin, and cholesterol-dependent cytolysin families. The hemolysin family includes α-hemolysin, γ-hemolysin AB, and leukocidins secreted by *S. aureus*. These PFTs contribute to *S. aureus* pathogenesis by modulating host immune response, killing immune cells, disrupting epithelial barriers, and altering intracellular signal transduction [39]. All staphylococcal PFT components have a similar fold, which is also shared by the *C. perfringens* necrotic enteritis toxin B, *Vibrio cholerae* cytolysin [40,41], and cytotoxin K (CytK) [42] and Hemolysin II (HlyII) of *B. cereus* [43]. Interestingly, *B. cereus* HlyII distinguishes from the other PFTs by a structurally unique C-terminal domain [44]. The function of this HlyII C-domain is currently unknown. The aerolysin family includes aerolysin produced by Gram-negative *Aeromonas* spp., the α-toxin produced by *Clostridium septicum*, monalysin produced by *Pseudomonas entomophila,* and parasporins produced by *Bacillus thuringiensis*. In pathogens such as *Aeromonas hydrophila*, aerolysins contribute to bacterial dissemination, possibly through disruption of epithelial barriers. [45]. The family of thiol activated, cholesterol-dependent cytolysins (CDC) is a prominent family of bacterial toxins. Members of this family originate from more than 20 Gram-positive species belonging to the *Bacillus, Clostridium, Streptococcus,* and *Listeria* genera [46,47]. The contribution of CDCs to eukaryotic cell infection depends on pathogens. For example, listeriolysin O (LLO) mediates intracellular survival of *Listeria* in various eukaryotic cell [48], perfringolysin O (PFO) mediates gas gangrene in *C. perfringens* infections [49], and pneumolysin (Ply) contributes to tissue damages caused by *Streptococcus pneumonia*, anthrolysin O (ALO) prolongs *Bacillus anthracis* survival in mice [50], and cereolysin O (HlyI, CLO) produced by *B. cereus* is lethal when injected intravenously into mice [51,52].

Until now, some PFTs family remain unclassified orphans, such as the repeats-in-toxin (RTX) family of proteins, which are widespread amongst bacteria [39]. Members of the RTX family exhibit two common features. First, their primary polypeptide sequence contains repetitions of glycine- and aspartate-rich sequences, typically nonapeptides, which are located in the C-terminal portion of the protein. Second, they are secreted via the type I secretion system. RTX toxins are secreted by a variety of Gram-negative pathogens (*Escherichia*, *Proteus*, *Pasteurella*) and form ion-permeable pores in several eukaryotic cell types, such as immune cells, epithelial cells, or erythrocytes [53]. The model of the RTX family is the α-hemolysin of *E. coli* [54].

### 2.3. AB Toxins

AB toxins differ in their primary structure, size, structural organization (monomers, oligomers), specificity for the target cells, as well as their biological effects. However, they are all constituted of two molecular components, A and B, topologically distinct. Binding of the B component to the eukaryotic cell surface is a prerequisite to the internalization of the AB complex by endocytosis [55]. In host cell, the component A of ADP-ribosylating AB toxins catalyze the transfer of ADP-ribose from NAD to a variety of eukaryotic proteins, including Rho proteins (*C. botulinum* C3 exoenzyme), heterotrimeric G proteins (*V. cholerae* cholera toxin and *B. pertussis* pertussis toxin) and actin (*C. botulinum* C2 toxin) [56]. The component A of non-ADP-ribosylating AB toxins works differently. For example, the Shiga toxin component A, produced by *Shigella dysenteriae*, cleaves host cell rRNA and prevents the attachment of charged tRNAs, thus stopping protein synthesis in target cells. The anthrax toxins, produced by *B. anthracis* [57], is composed of two different A-components known as Lethal Factor (LF) and Edema Factor (EF), which share a common B-component, known as protective antigen (PA). LF is a metalloproteinase that inhibits mitogen-activated kinase-kinase. EF is an adenylate cyclase that catalyzes the production of a large amount of cAMP in host cells. This most likely impairs host defenses.

### 2.4. Toxins Injected via Secretion Systems

Exotoxins can be directly injected into the target cell cytoplasm by secretion systems of type III, IV, and VI (T3SS, T4SS, T6SS). T3SS is a complex needle-like nanomachine that is triggered when a pathogen comes in close contact with host cells. The injected exotoxins cause changes in target cell function, which facilitate the pathogen′s ability to survive and replicate [58]. The best-studied bacterial pathogens that use T3SSs to inject effector exotoxins are *Pseudomonas aeruginosa*, *Yersinia pestis*, and enteropathogenic bacteria, which cause foodborne illnesses. Enteropathogens (*Yersinia* spp., *Salmonella* spp., *Shigella* spp., and EPEC) produce distinct syndromes, ranging from diarrhea to systemic fever-like typhoid because their exotoxins target different host cells and molecules [59]. T4SSs are structurally very different. In gram-negative bacteria, T4SSs spans the entire cellular envelope and comprises ATPases, pilus proteins, and translocation channel proteins. It has been shown recently that the opportunistic human pathogen *Stenotrophomonas maltophilia* uses a T4SS to inject toxins into target bacteria [60]. T6SS is a membrane-spanning machine that resembles a bacteriophage tail-structure. It is used by many pathogens to inject toxins into both prokaryotes and eukaryotic cells [61,62,63].

## 3. Next-Generation Proteomics, a Powerful Toolbox to Study Bacterial Toxins

Major challenges to date consist of defining how toxins affect differentiated host cell types to define their contribution to the various steps of the infection process. For this, monitoring their production during the various steps of infection and defining toxin mechanisms, as described above, are of utmost interest. Next-generation proteomics which has numerous facets in terms of methodologies constitutes an excellent toolbox for the identification, quantification, and site localization of post-translational modifications of toxins. This powerful technology should help to tackle new challenges to improve our understanding of toxinogenesis.

### 3.1. Proteomics Potential for Toxin Detection 

Many reports describe proteomics approaches for the detection of bacterial toxins. In this review, we took the example of *B. cereus* to show how proteomics, and specifically exoproteomics [12], i.e., the analysis of the proteins present outside of the cells, can advance knowledge of the toxinogenic potential of a bacterial strain.

**Figure 2:**
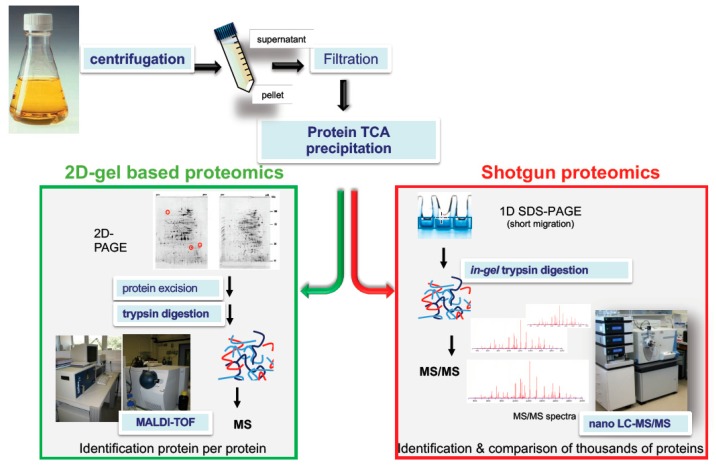
Examples of proteomics-based strategies to decipher the toxigenic profile of a bacterial pathogen. Pathogens are grown in regulated batch cultures that mimic the conditions encountered in the host. Exoproteins are collected by centrifugation of the culture medium and then filtration (0.22 µm) of the resulting supernatant. Exoproteins are precipitated using trichloroacetic acid (TCA) or collected by other methods. In two-dimensional (2D) gel–based proteomics, exoproteins are resolved by 2D gel electrophoresis. Exoproteins of interest are excised from the gels and digested using trypsin. Tryptic peptides are analyzed on a Matrix Assisted Laser Desorption Ionisation-Time of Flight (MALDI-TOF) mass spectrometer. Protein identification relies on the comparison of the measured mass of the tryptic peptides with the predicted masses of tryptic peptides from database protein sequences. This approach is quite time consuming as mass spectrometry measurement should be done on each protein spot. In shotgun proteomics, the whole proteome is collected as a single band from a one-dimensional SDS-Polyacrylamide Gel Electrophoresis (1D SDS-PAGE) gel, which is treated and in-gel proteolyzed with trypsin. Alternatively, the proteins may be in-solution proteolyzed in a gel-free approach. The resulting peptide mixture is injected into a reverse phase chromatography column coupled to a high-resolution mass spectrometer. The recorded tandem mass (MS/MS) spectra are processed against a protein sequence database using a search engine such as Mascot Daemon algorithm (Matrix Science). Exotoxin semi-quantification is simply but reliably evaluated by MS/MS spectral counts.

**Figure 3:**
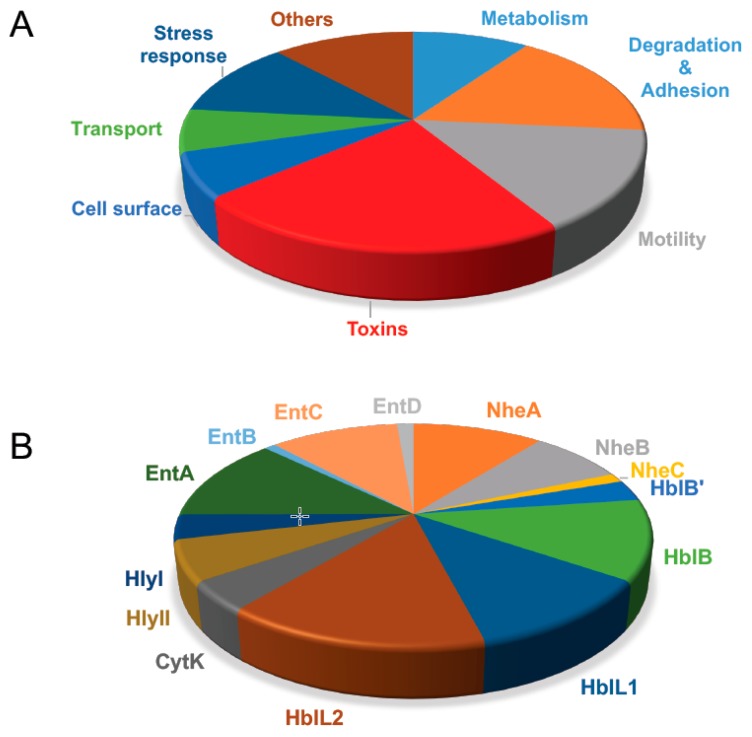
Relative abundances of *B. cereus* ATCC 14579 proteins in culture supernatant. The relative abundances were determined by averaging the Normalized Spectral Abundance Factor (NSAF) values of three biological samples harvested at the early exponential growth phase [65]. The diagrams represent the average abundance of (**A**) each functional protein group and (**B**) each protein belonging to the toxin group. CytK (BC1110): Cytotoxin K; NheA (BC1809), Non-hemolytic enterotoxin, lytic component A; NheB (BC1810): Non-hemolytic enterotoxin, lytic component B; NheC (BC1811): non-hemolytic enterotoxin, component C; HblB’ (BC3101): Hemolysin BL, putative binding component B′; HblB (BC3102): Hemolysin BL, binding component B; HblL1 (BC3103): Hemolysin BL, lytic component L1; HblL2 (BC3104): Hemolysin BL, lytic component L2; HlyII (BC3523): Hemolysin II; HlyI (BC5101): Cereolysin; EntA (BC5239): enterotoxin-like; EntB (BC2952): enterotoxin-like; EntC (BC0813): enterotoxin-like; EntD (BC3716): enterotoxin-like.

The first proteomics study that identified *B. cereus* exotoxins was performed by Gohar and collaborators [64], who investigated the impact of the PlcR regulon on the secreted proteins of *B. cereus* by comparing the extracellular proteomes of a *plcR* mutant and its parental strain ATCC 14579. In this pioneering work, proteins were extracted from culture supernatants at the onset of the stationary phase, precipitated and resolved by electrophoresis on two-dimensional (2D) gels. Among the ~500 protein spots detected, 23 protein spots that showed intensity differences between the two strains were excised, proteolyzed with trypsin, and analyzed by peptide mass fingerprint using MALDI-TOF mass spectrometry (Figure 2). Six proteins were identified as toxins and PFT-components. These proteins were HlyI, CytK, the NheA and NheB components of Nhe, and the Hbl-B and Hbl-L2 components of Hbl. All of these proteins are abundant proteins within the *B. cereus* exoproteome and were found members of the PlcR-regulon. Taking advantage of the methodology developed in 2002, Gohar and collaborators then investigated the differences in the exoproteomes for strains of the three species of the *B. cereus* group devoid of toxin-bearing plasmids: *B. cereus* ATCC 14579, *B. anthracis* 9131 strain, and *B. thuringiensis* 407 Cry^-^, from cultures in early stationary phase [65]. Forty-six proteins were identified in the *B. cereus* exoproteome including the Hbl -L1, L2, and B components, the NheA and NheB components, Cyt K, and HlyI. Hbl, Nhe, and CytK were also identified in the *B. thuringiensis* exoproteome, together with the thuringolysin Tlo (that corresponds to HlyI) and HlyII, indicating that exoproteomics is an effective method to (i) establish the toxinogenic profile of a *B. cereus sensu lato* strain and to (ii) study the variability existing in the toxinogenic profile of related strains. A third proteomics study confirmed these findings [66]. In 2010, Clair and collaborators [67] considerably expanded on the inventory of *B. cereus* by analyzing its exoproteome at early exponential growth phase, under conditions considered to mimic those encountered in the small intestine with a shotgun approach and the use of a tandem mass spectrometer incorporating an Orbitrap analyzer (Figure 2). They showed with a proteomic label-free quantitative approach that (i) toxins and putative toxins represented approximately 20% of exoproteome, and (ii) it was possible to detect the third component of enterotoxin Nhe (NheC) and the putative fourth component of Hbl (HblB’), which have never been detected before (Figure 3). NheC accumulated in the extracellular medium in a much lower amount than NheA and NheB, regardless of the growth phase [67,68,69]. This is in agreement with the literature data reporting that a molar ratio of 10:10:1 (NheA:NheB:NheC) is important for maximum toxicity [70,71]. HblB’ also accumulates in a lower amount than L2, L1 and B components of Hbl (Figure 3). HblB’ is the product of the *hblB* gene, which is located 376 bp downstream of the *hblCDA* cistron encoding the L2, L1 and B components of Hbl (Figure 1). It has not yet been determined whether it is an accessory protein or an active protein of the Hbl PFT. In addition to well-studied toxins, *B. cereus* secretes four putative enterotoxins EntA, EntB, EntC, and EntD, which accumulated differently in the extracellular media (Figure 3). While EntA, EntB, and Ent C were detected by shotgun proteomics, EntD was detected by proteogenomics [67,72]. Although it has not been yet proven that these four proteins are true enterotoxins, it was shown that EntD is clearly involved in *B. cereus* virulence. Today, the complete genome sequences of a large number of *B. cereus* strains are publicly available in reference databases. With such information in hand, it is now possible to identify up to a thousand proteins in the exoprotome of *B. cereus*, against 26 at the very beginning of the proteomic era. Among these proteins, there are several cytosolic proteins that could be exported by a so-far unknown mechanism [73,74], toxins and other secreted proteins, but also several proteins of unknown functions. Whether new uncharacterized toxins are among these unknown proteins is an open question. Only functional studies would answer this question. However, these proteomic results paved the way for more detailed studies on toxinogenesis. Differential proteomics also allowed to track global changes of the toxin repertoire under changing conditions and upon alteration of particular gene expression [75,76,77,78].

Proteomics describes the abundance of molecular players, directly explaining the phenotype of the microorganism. Compared with genomics and transcriptomics, this approach has the advantage of defining proteins that are differentially produced, not just purely transcriptionally regulated. Also, it can define proteins that are differentially located or secreted outside of the cell (i.e., to the media or at the surface). In many cases, the proteins that fall into these categories can be predicted from their sequences directly obtained by genome sequencing, but proteomics is quite able to show the presence of some unexpected proteins (unpredicted). Furthermore, only proteomics can detect post-translational modifications of proteins.

### 3.2. Proteomics Analyses for Detection of Post-Translational Modifications (PTMs)

**Figure 4:**
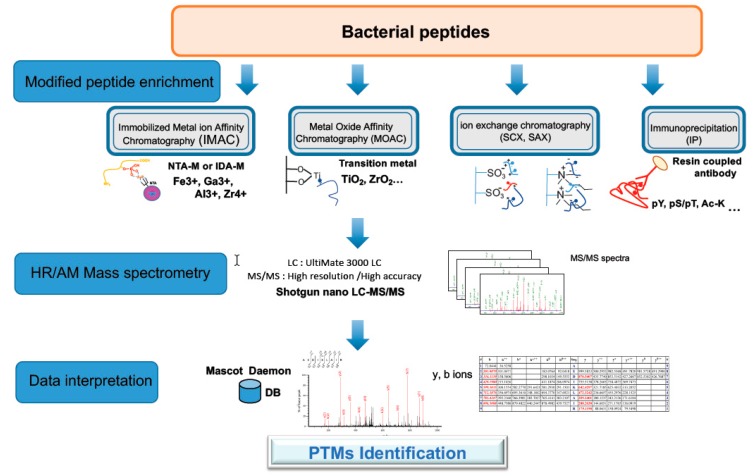
Strategy for phospho- and acetyl-proteomic analysis. The strategy for Post-Translational Modification (PTM) proteomics of bacterial proteins involves protein extraction, trypsin digestion of proteins, enrichment of PTM peptides using an appropriate method (here four methods are indicated), nano LC MS/MS analysis of the enriched PTM peptides, peptide identification, mapping PTM sites, and quantification. The most common methods for phosphopeptide enrichment and acetylpeptide enrichment are TiO2 chromatography and immunoenrichment, respectively. High-resolution tandem mass spectrometry is the most appropriate detection method as the site of modification can be delineated with precision.

For a long time, PTMs have been considered to be restricted to eukaryotes. During the last decade, advanced detection methods of PTMs, including enrichment of the modified peptides combined with high-resolution mass spectrometry, were applied to numerous microorganisms (Figure 4). The results highlighted that bacteria have also developed a large arsenal of PTMs [79,80,81]. The enzymatic arsenal for creating these modifications can be identified in their genome sequences. Bacteria contain many different types of PTMs, including those commonly present in eukaryotes, such as oxidation, phosphorylation, acetylation, and S-thiolation [82]. PTMs can affect both homeostasis of proteins and the cellular processes in which they are involved [83]. For a certain protein, diverse PTMs can be present in combination, and their on–off states vary under different conditions, thereby fine-tuning their activities, localizations, and interactions with other proteins. These modifications may potentially affect toxins and modulate their function and thus are important to study.

### 3.3. Protein Oxidation

Oxidation of the thiol group of cysteine and methionine residues in proteins has emerged as a widespread and important post-translational modification. Methionine and cysteine are readily oxidized by many of the reactive oxygen species (ROS) generated in biological processes [84,85]. The modification of cysteine by cellular ROS can generate a number of chemical products, including reversible sulfenic acid (SOH) modification and more stable sulfinic (SO_2_H) and sulfonic (SO_3_H) modifications, only a subset of which can be currently detected with good sensitivity in proteomes [86]. As a consequence, our understanding of the full spectrum of oxidation-sensitive cysteine in bacterial human pathogen proteomes remains incomplete. The impact of cysteine oxidation on the biological activity of exotoxins is probably small because the number of cysteine residues in secreted proteins is low, especially in gram-positive bacteria [87]. However, it was reported that *B. cereus* HlyI has an activity depending on the oxidation/reduction state of its cysteine residue [88].

Oxidation of Met generates methionine sulfoxide (MetO) that can be detected readily by mass spectrometry through a mass increase of 15.9949 atomic mass unit. The formation of MetO is reversible and MetO reduction is catalyzed by methionine sulfoxide reductases (Msr) [89]. In the case of *B. cereus*, shotgun proteomics allowed large-scale and high-throughput identification of MetO proteins [68]. Interestingly, analysis of *B. cereus* exoproteome showed that (i) Nhe and Hbl enterotoxins contained oxidizable methionines, (ii) the redox state of Met residues in toxins was regulated by MsrAB and, (iii) Met residues in enterotoxins could act as ROS scavengers [69]. However, the effect of Met oxidation on the biological activity of enterotoxins have not been yet analyzed. 

### 3.4. Protein S-Thiolation

The redox-sensitive proteome can be post-translationally modified through disulfide linkages between glutathione (GSH), bacillithiol (BSH) or mycothiol and redox-sensitive cysteine residues within proteins [90,91,92]. During the last years, many targets for protein S-thiolation have been discovered through shotgun proteomics and quantitative thiol-redox proteomics. Most of these targets are antioxidant and metabolic enzymes. CDCs are also S-thiolation targets, as shown for LLO of *Listeria monocytogenes*. Importantly, S-glutathionylation of LLO was reported to be necessary for its optimal activity [93].

### 3.5. Protein Phosphorylation

Phosphorylation consists in the reversible covalent addition of a phosphate group, from the phosphate donor ATP to specific residues of a target protein, the most frequent being hydroxyl groups of serine, threonine or tyrosine residues. Histidine, arginine, lysine, and cysteine residues can also be phosphorylated but to a lesser extent. In contrast to eukaryotes, the extent and biological function of protein phosphorylation in bacteria are poorly defined. One explanation is that protein phosphorylation in bacteria is dramatically lower than in eukaryotes, making phosphoproteomics analyses challenging. However, several bacterial phosphoproteomes have been successfully characterized including some human pathogens, *Helicobacter pylori* [94], *Staphylococcus aureus* [95], *L. monocytogenes* [96], and EPEC [97]. Together, these studies provide excellent bases for further investigations on regulatory mechanisms involved in pathogenicity/virulence. To our knowledge, few phosphoproteomics studies focused on the extracellular compartment. Ouidir and collaborators explored the phosphoexoproteome of *P. aeruginosa* [98]. They highlighted 28 secreted virulence factors with various phosphorylation sites, confirming the important role of this PTM on virulence. To our knowledge, no phosphotoxins was detected using phosphoproteomics.

### 3.6. Protein Acetylation

Protein acylation can be defined as the transfer of an acyl group from a convenient biochemical donor molecule to an amino group on a protein. Many proteins can be acylated by activated acyl groups such as acyl-CoAs and acyl-phosphates [99,100]. Acetylation can occur either chemically (non-enzymatically) or enzymatically. Based on the chemical nature of the acetylated amino group, two types of protein acetylation can be considered, each one exhibiting specific characteristic features. The acetylation of the α-amino group of the N-terminal amino acid of proteins is possible in some bacteria, which was previously considered to be very rare [101,102]. In contrast, the acetylation of proteins at the ɛ-amino group of internal lysine residues is a widely distributed PTM. Until recently, few bacterial proteins were known to be acetylated. However, the increasing power of high throughput proteomic techniques changed this view [103,104,105]. Acetylome analysis in bacteria reveals that acetylation occurs on diverse proteins involved in various metabolic pathways but also in virulence [106]. Interestingly, in *P. aeruginosa*, some exotoxins such as exotoxin A and hemolysin were reported as being acetylated [107]. The biological role of lysine acetylation remains unclear.

### 3.7. Emerging Topics

Proteomics approaches showed that acetylation was as common as phosphorylation in bacterial proteins. This entertains the possibility that these two PTMs could both have a role in the function of the modified proteins. Proteomics approaches also showed that lysine side chain can be modified by a variety of chemical groups such as acetylation and succinylation but also butyrylation, crotonylation, dimethylation malonylation, methylation, propionylation, and trimethylation. Intriguingly, Gaviard et al. [108] showed that some lysines of two secreted virulence factors were modified by these nine different PTMs in *P. aeruginosa*, leading to the emerging concept of cross-talk between PTMs in bacteria. This concept is further supported by biological follow-up studies that are starting to reveal bacterial proteins and processes regulated by multiple modifications [109]. Hence, studying all PTMs and making sense of the countless interactions between them is presently a daunting task but constitutes a challenge. The continuous development of new computational methods for exploiting deep proteomics recorded data could help in the near future to explore in more details potential PTMs of proteins involved in toxinogenesis.

## 4. Proteogenomics, Metaproteomics, and New Tools for a More Integrated Vision of Bacterial Toxins

The advent of genomics resulted in a considerable amount of genome data and expanded our knowledge on the potential of microorganisms to synthesize toxins. While sequencing new genomes is today trivial, their structural and functional annotation is relying on bioinformatics programs and previous molecular biology knowledge. Several types of annotation errors may be found in such automatic genome annotation, such as misidentification of a set of genes, wrong identification of the correct translation start, and incorrect assignation of the coding DNA strand or reading frame [110]. Peptide information obtained by tandem mass spectrometry may help to establish the presence of a given gene encoding a new polypeptide or better define the translation initiation site of numerous proteins [111]. The integration of proteomics and genomics data collected on the same microorganisms is of interest for detecting such errors and improving the genome annotation quality [112,113], especially for rare taxonomic phyla [114]. Specific proteogenomic experiments could be performed in the future for exploring whether toxin-producing microorganisms are well genome annotated and further characterizing their toxin potential. For this, multiplying the growth conditions for the pathogens, eventually in presence of the host, would be advantageous for a more comprehensive proteogenomic re-annotation. Another trendy approach is metaproteomics, which aims at identifying the proteins of complex samples encompassing numerous microorganisms [115,116]. Currently, samples containing more than a hundred microorganisms can be scrutinized for their proteome content with a shotgun strategy. Ideally, for interpreting the large MS/MS dataset acquired with a high-resolution instrument, a database constructed from metagenomics data obtained from the same sample is required. In such analysis, proteins specifically produced by a microorganism in contact with others are of special interest. Probably, specific toxins could be detected and correlated with the presence of specific microorganism neighbors.

As illustrated in several reviews [117,118,119], proteomics outcomes are highly dependent on the tandem mass spectrometer used. While new generations of instruments are proposed almost every year with improved performances in terms of sensitivity, resolution, and speed, increased coverage of the proteome is expected in terms of the number of proteins and their sequence coverage, as well as a more precise quantitation of these proteins. Currently, an instrument such as a Q-Exactive HF tandem mass spectrometer which is considered as a workhorse for proteomics allows us to identify up to 2000 proteins from a bacterial extract in a single 90 min run [120,121] and up to 4000 from a yeast extract [122]. The most recent tandem mass spectrometers allow microbiologists entering in the complete proteome era.

Furthermore, new strategies in proteomics have recently emerged for improving the sensitivity of the methodology and the quantitation of proteins. Most studies have been conducted until now in data-dependent acquisition (DDA) mode, where the MS/MS acquisition is done on the fragments arising from the fragmentation of a single peptide species after identification of its molecular mass and charge. Targeted proteomics via selected reaction monitoring (SRM) or multiple reaction monitoring (MRM) is known to be a more sensitive method than label-free shotgun proteomics. In this case, specific fragment ions arising from the fragmentation of a given peptide are monitored, and their signals are compared to the signals of synthetic peptide standards analyzed in the same condition. The parallel reaction monitoring (PRM), which is an alternative method of targeted quantification, available for quadrupole-Orbitrap hybrid instruments, was shown to outperform DDA in terms of reproducibility and detection efficiency for the phosphoproteome analysis of a bacterium [123]. A new acquisition mode is also available for several years, the data-independent acquisition mode (DIA), where the MS/MS acquisition is done on the fragments arising from all the co-eluting peptide species [124]. In this case, the MS/MS spectrum is a composite spectrum and the identification of the peptides is more challenging. However, the identification and quantitation of new peptide species can be performed by iterative search on the previously recorded signal. Such improvement may lead to a gain in sensitivity and a better assessment of toxins in complex samples.

## 5. Concluding Remarks and Future Perspectives

The recent developments in proteomics, in mass spectrometry instrumentation and bioinformatics tools, have revolutionized toxin research, as illustrated with the large panel of results obtained on *B. cereus*. Proteomic approaches allow rapid, precise, and large-scale analysis, providing in-depth view of the toxigenic profile of any pathogenic strain. Further exploration of proteomes should reach comprehensiveness and could be applied to detailed kinetics for numerous clinical strains to fully understand the relationships between their protein repertoire and their pathogenicity power. Large-scale proteomic PTM characterization of these pathogens is challenging but worth investigating.
